# Micromachined Fluid Inertial Sensors

**DOI:** 10.3390/s17020367

**Published:** 2017-02-14

**Authors:** Shiqiang Liu, Rong Zhu

**Affiliations:** State Key Laboratory of Precision Measurement Technology and Instrument, Department of Precision Instruments, Tsinghua University, Beijing 100084, China; liusq13@mails.tsinghua.edu.cn

**Keywords:** micromachined fluid inertial sensor, gyroscope, accelerometer, thermal convection, thermal expansion, jet flow, thermal flow

## Abstract

Micromachined fluid inertial sensors are an important class of inertial sensors, which mainly includes thermal accelerometers and fluid gyroscopes, which have now been developed since the end of the last century for about 20 years. Compared with conventional silicon or quartz inertial sensors, the fluid inertial sensors use a fluid instead of a solid proof mass as the moving and sensitive element, and thus offer advantages of simple structures, low cost, high shock resistance, and large measurement ranges while the sensitivity and bandwidth are not competitive. Many studies and various designs have been reported in the past two decades. This review firstly introduces the working principles of fluid inertial sensors, followed by the relevant research developments. The micromachined thermal accelerometers based on thermal convection have developed maturely and become commercialized. However, the micromachined fluid gyroscopes, which are based on jet flow or thermal flow, are less mature. The key issues and technologies of the thermal accelerometers, mainly including bandwidth, temperature compensation, monolithic integration of tri-axis accelerometers and strategies for high production yields are also summarized and discussed. For the micromachined fluid gyroscopes, improving integration and sensitivity, reducing thermal errors and cross coupling errors are the issues of most concern.

## 1. Introduction

Micromachined inertial sensors have been developed for decades and are gradually becoming mature with the advancement of Micro-Electro-Mechanical System (MEMS) technology. Generally speaking, micromachined inertial sensors include accelerometers used for measurements of linear acceleration, velocity and position or tilt angle, shock, jerk transduction, and gyroscopes used to measure the angular rate of moving objects [1]. Thanks to their small size, batch fabrication, and low cost, micromachined inertial sensors play important roles in civil and military applications, e.g., smartphones, wearable equipment, motion-tracking equipment, vehicle navigation, unmanned aerial vehicle, virtual-reality (VR) equipment, new generation of motorcycle ABS systems, etc. [2,3,4,5]. 

Micromachined fluid inertial sensors are an important class of inertial sensors. They use a gas (e.g., He, air, SF_6_) [6] or a liquid (e.g., oil, alcohol, water) [7,8,9] as the moving and sensitive elements instead of a solid mass. Considering the fact that gases are more commonly used than liquids, these sensors are usually called micromachined gas inertial sensors as well. Conventional silicon and quartz inertial sensors need a solid mass and therefore suffer from fragility, complex fabrication processes and squeezed-film air damping [10]. Obviously, the fluid inertial sensors without a solid proof mass, having advantages of simple structures, low cost, high shock resistance [11,12,13], large measurement ranges [10,14], and smaller scatter of operational parameters in a production batch, have demonstrated attractive commercial prospects. Despite these advantages, fluid inertial sensors are facing challenges of sensitivity, bandwidth, and susceptibility to ambient temperature, etc.

There are mainly two kinds of micromachined fluid inertial sensors: thermal accelerometers and fluid gyroscopes. The micromachined thermal accelerometer is based on the free-convection heat transfer of a tiny hot air bubble in an enclosed chamber, and was firstly reported in the 1990s by Leung et al. [14]. Since then, many studies have been conducted on these sensors. Most of the studies have focused on improving the sensor performance by optimizing the fluid medium with proper thermal properties (e.g., thermal conductivity, thermal diffusivity, kinematic viscosity), temperature sensing (e.g., thermal couple, thermistor), the material of the thermistor (e.g., metal thermistor, polysilicon thermistor, silicon PN junction thermistor) and the structure of the sensor (e.g., cavity shape and size, cap shape and size, distribution and size of the heater and thermistors) [6,7,8,9,10,11,15,16,17,18,19,20,21,22,23,24,25,26,27,28,29,30,31,32,33,34,35,36,37,38,39,40]. Combinations of Finite-Element-Modeling (FEM) simulation and experimental validation are the most commonly-used research methods. The signal conditioning circuits were also investigated to improve the performances of the accelerometers [41,42,43,44]. In addition, the fabrication processes were optimized to ensure their compatibility with the mature micromachining process. All these efforts resulted in improvements of the accelerometers (such as higher sensitivity, lower noise level, higher bandwidth and lower power consumption), monolithic multiaxial sensors and better manufacturability for batch production [10,45,46,47,48,49,50,51,52,53]. Modeling and simulation technology have achieved great progress in the past decades. More accurate behavioral models were established for guiding and evaluating subsequent design [22,25,26,27,28,41,49,54,55,56]. The establishment of the Hardware Description Language (HDL) model of the accelerometer made it possible to design the sensor and Application Specific Integrated Circuit (ASIC) simultaneously at the system level [56,57]. Moreover, test and calibration strategies for manufacturing micromachined thermal accelerometers with a fully electrical setup were proposed and optimized, and many electrical test methods and alternative test methods based on the high-level behavioral model and fault model were proposed as well. Other studies focused on decreasing the time cost in product screening and increasing production yield for batch production of accelerometers, which is very helpful to decrease the final cost of the products and make them more competitive in the market [57,58,59,60,61,62]. Applications of thermal accelerometers have been also investigated, such as convection sensors used for tilt measurement [6].

Micromachined fluid gyroscopes use a fluid as proof mass. In terms of their driving principles, two kinds of micromachined fluid gyroscopes have been reported, namely jet flow gyroscopes and thermal gas gyroscopes. The jet flow gyroscope uses a laminar gas flow driven by a micro pump, while the thermal gas gyroscope uses a flow induced by thermal convection or thermal expansion. The micromachined jet flow gyroscope was firstly proposed by Ding and Zhu et al. in 2001 [63]. Many studies have been conducted to improve their performance by optimizing their structures, materials and working conditions [10,64,65,66,67,68,69,70,71,72,73,74,75,76,77,78]. The present research of the micromachined jet flow gyro focused on monolithic integration of flow detectors and micro pump in the premise of maintaining high flow velocity [78,79,80,81,82,83,84,85,86,87,88,89,90,91,92]. The first thermal gas gyroscope was proposed by Zhu et al. in 2001 [93] and prototyped in 2005 [71,72], the present work focused on suppression of its obvious cross coupling with acceleration [73,94,95,96,97,98,99,100,101,102]. Thermal compensation was also studied for robust temperature performance [103]. Compared with thermal accelerometers, the micromachined fluid gyroscopes are less mature and still developing, although more and more advances are emerging and great promotion should be achieved in the near future.

## 2. Working Principles of Micromachined Fluid Inertial Sensors

### 2.1. Micromachined Thermal Accelerometers

**Figure 1:**
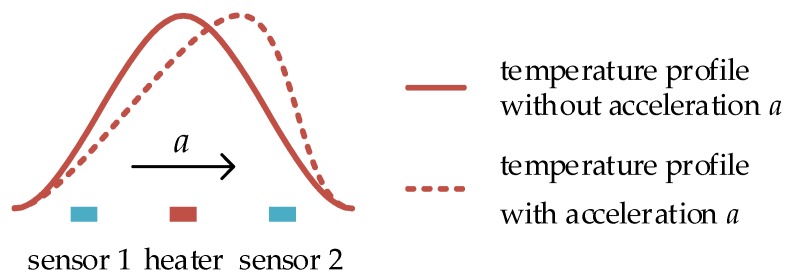
Operation principle of micromachined thermal accelerometer.

The micromachined thermal accelerometer is based on free-convection principle. As illustrated in Figure 1, a heater is located in the center and two temperature sensors are placed at two sides of the heater. The heater is electrically heated to a higher temperature and heats up the surrounding gas or liquid and lowers the medium density. Free-convection is induced and the temperature profile produced by the heater is symmetrical at the absence of lateral acceleration (shown as the solid curve in Figure 1). In this case, the local temperatures at the two temperature sensors are equal. While, at the presence of lateral acceleration, the temperature profile is deflected (shown as the dashed curve in Figure 1), the temperature difference between two sides of the heater is measured by the two temperature sensors and is proportional to the lateral acceleration. By this sensing principle, the sensor transduces the lateral acceleration to the temperature difference which is read out by the subsequent conditioning circuit, and therefore the sensor is used as an accelerometer [14]. 

In a micromachined thermal accelerometer, the heater operated at a higher Joule power is a thermal resistor made of metal or polysilicon, while the temperature sensors are operated at lower Joule powers to minimize their disturbances on the temperature field. To detect the change of the temperature profile, two kinds of temperature sensors can be used, the first one is thermistors made of metal or polysilicon, and the other is thermopiles. For a thermistor sensor, the most important parameter is the temperature coefficient of resistance (TCR) which dominates the sensitivity of the accelerometer. For thermopiles, the Seebeck coefficient dominates the accelerometer sensitivity. Although the Seebeck coefficient of semiconducting thermoelectric materials is usually much larger than those of metals, metal thermopiles are most commonly used in micromachined thermal accelerometers due to their compatibility with micromachining of the accelerometers. Raising the heater temperature can improve the sensitivity, but the power consumption increases as well. In addition, the properties of the surrounding fluid have a complex influence on the accelerometers. For example, a fluid with high thermal diffusivity contributes to a high bandwidth but may lead to a low sensitivity. In addition, the structure of the accelerometer also greatly affects its performances, such as the shapes of the heater and temperature detector, the distance between them, the shape and size of the chamber, etc. Therefore, optimization of the structure is very important issue in design of a thermal accelerometer.

### 2.2. Micromachined Fluid Gyroscopes

#### 2.2.1. The Jet Flow Gyroscope

**Figure 2:**
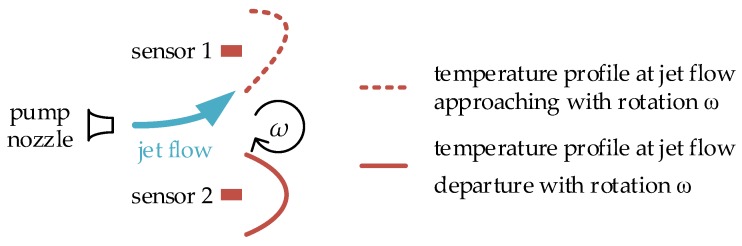
The schematic of jet flow gyroscope.

A jet flow gyroscope is based on a cool jet flow generated by a mechanical pump, and two anemometers (sensor 1 and sensor 2 in Figure 2) symmetrically placed on the two sides of the nozzle are used as flow sensors to detect the jet flow. As shown in Figure 2, at the absence of rotation, the jet flow goes straight, and the flows passing through two flow sensors are the same, while, in the presence of rotation, the jet flow is deflected due to the Coriolis acceleration. The flows passing through the flow sensors on the two sides are different and detected by the sensors. If a hot-film or hot-wire is used as the flow sensor, which is self-heated and serves as both a Joule heater and a temperature detector at the same time, the resistance of the sensor corresponds to the forced convective cooling induced by the jet flow passing through. The resistance difference between two sensors is then converted to an electrical signal by the subsequent conditioning circuit which is used to deduce the rotation rate [64]. 

TCR of the thermal flow sensors has an important effect on the gyroscope sensitivity, and thermal resistance material with a high TCR is preferred to achieve a high sensitivity. Meanwhile, the heating of the thermal flow sensors suffers from thermal stress, therefore the structure must be optimized to minimize the corruption induced by the thermal stress. The layout of the flow sensors and the pump nozzle also have an influence on the gyroscope performance and should be designed properly. According to the working principle mentioned above, jet flow velocity is another key factor of the jet gyroscope, a higher flow velocity implies a higher sensitivity. Therefore, the most important consideration of a monolithic jet flow gyroscope is to improve the integrated micro-pump to achieve stable and high-speed fluid flow. 

#### 2.2.2. The Thermal Gas Gyroscope

**Figure 3:**
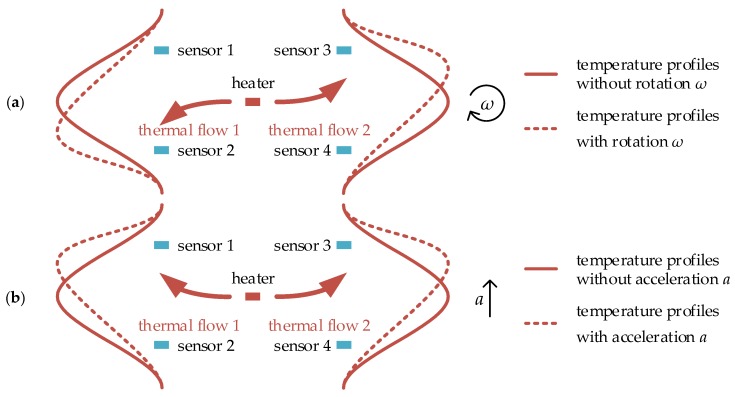
The schematic of thermal gas gyroscope, (**a**) temperature profiles deflected by Coriolis acceleration and (**b**) temperature profiles deflected by linear acceleration.

The thermal gas gyroscope is based on thermal flow induced by thermal convection or thermal expansion. In the gyroscope, the heater is electrically heated to a higher temperature and used to generate thermal flow and form a temperature profile as shown in Figure 3. Temperature sensors symmetrically placed on two sides of the heater are used to detect the skewness of the temperature profile induced by the rotation. At the absence of rotation, the thermal flow goes straight, and the temperature profile is symmetrical as illustrated by the solid curve in Figure 3a, whereas in the presence of rotation, the thermal flow is deflected due to the Coriolis acceleration, which results in the skewness of the temperature profile illustrated by the dashed curve in Figure 3a. The temperature difference caused by the temperature profile skewness is measured by the distributed temperature sensors, and is proportional to the rotation rate. 

Besides rotation measurement, the thermal gas gyroscope is also sensitive to a linear acceleration when gauging the rotation. As shown in Figure 3b, the two opposite thermal flows and temperature profiles on the two sides of the heater are deflected in the same direction in the presence of a linear acceleration *a*. However, in the presence of rotation *ω*, the two opposite thermal flows and temperature profiles are deflected in the opposite direction due to the opposing Coriolis accelerations, which is shown in Figure 3a. Therefore, compared with the thermal accelerometer, which needs one pair of temperature sensors to detect linear lateral acceleration (shown in Figure 1), two pairs of temperature sensors are essential for the thermal gyroscope to detect the skewness of the temperature profiles on the two sides of the heater to extract the rotation and linear acceleration, and thereby both rotation and acceleration can be detected simultaneously.

To overcome the cross-coupling issue between the rotation and acceleration measurements in thermal gas gyroscopes based on buoyancy, the thermal expansion-based gyroscope was proposed [96,97]. In a thermal expansion-based gyroscope, it is essential to alternately heat and cool the heaters of the gyroscope to generate thermal expansion/contraction flow [96,97]. Thermistors or thermopiles can be used as the temperature sensors. High TCR for thermistors and high Seebeck coefficient for thermopiles are desirable to improve the gyroscope sensitivity, and the fabrication compatibility of the temperature sensor should be taken into account as well. Heating power needs to be set properly according to the trade-off of sensitivity and power consumption. In addition, fluid properties and structures of the thermal gas gyroscope should be optimized to achieve better performance.

## 3. Developments of Micromachined Fluid Inertial Sensors

### 3.1. Micromachined Thermal Accelerometers

Up to now, micromachined thermal accelerometers have been developed for about 20 years, and commercial products have entered the market since 2003 [104]. Many research institutes have investigated micromachined thermal accelerometers, such as Simon Fraser University, University Montpellier 2, University of Sfax, Hebei Semiconductor Research Institute, University of Minho, MEMSIC, etc. 

#### 3.1.1. Uniaxial and Dual-Axis Micromachined Thermal Accelerometers

**Figure 4:**
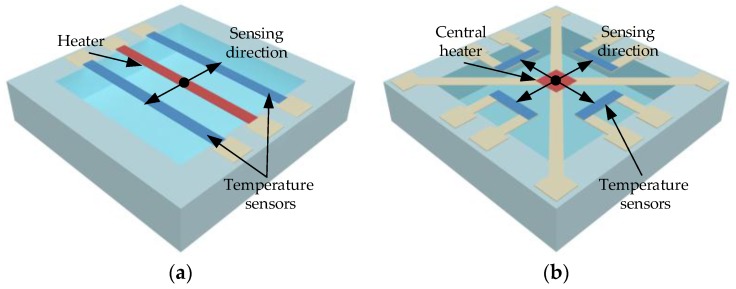
Illustrations of thermal convection accelerometer, (**a**) uniaxial accelerometer; (**b**) dual-axis accelerometer.

Early micromachined thermal accelerometers were mostly uniaxial or dual-axial sensors due to the fabrication limitations at that time. The uniaxial thermal accelerometer, whose working principle has been described in Section 2.1, is illustrated in Figure 4a. The dual-axial thermal accelerometer usually consists of a central heater and two pairs of temperature detectors orthogonally distributed along the two horizontal axes, which can measure the two orthogonal in-plane accelerations, as illustrated in Figure 4b.

Following the first uniaxial thermal accelerometer proposed by Leung et al. in 1997 [14], a dual-axis thermal accelerometer fabricated by similar fabrication process as an uniaxial accelerometer was proposed in 1998 [15]. The accelerometer has a very simple structure and requires only four masking steps in its fabrication. Compared with conventional silicon accelerometers, it is very simple, reliable, and inexpensive. After that, Leung et al. investigated the effects of heat power and fluid properties (pressure, Prandtl number and Rayleigh number) on the sensor sensitivity. N_2_, Ar, CO_2_, SF_6_ and other gas media under different pressure were used to improve the performance. 

Almost at the same time, Milanovi et al. from George Washington University proposed a convection-based accelerometer and tilt sensor implemented by a standard CMOS process. Temperature detectors using thermocouples and polysilicon thermistors were compared, and the results showed that the thermistors had better sensitivity (185 µV/g) than the thermocouples (115 µV/g) [36]. In 2001, Yang et al. from the Hebei Semiconductor Research Institute reported a micromachined convective accelerometer using polysilicon heater and thermistors. A sensitivity of 600 µV/g was achieved under an operating power of 87 mW, the response frequency was about 75 Hz and the corresponding noise equivalent acceleration was approximately 1 mg/Hz^1/2^ at 25 Hz [29]. In 2002, HSG-IMIT presented a thermal inclinometer based on free convective flow fabricated by SOI technology. SF_6_ was used as the fluid medium, presenting a high sensitivity of 6.6 mV/° under 45 mW power, a high resolution of 50 μg and a response time of 110 ms [6]. Technological Educational Institution (TEI) of Athens reported a CMOS-compatible thermal accelerometer, called porous silicon thermal accelerometer (PSTA), which used air as gas medium and a porous silicon (PS) layer assuring thermal isolation from the Si substrate. Thermopiles acted as temperature detectors, a sensitivity of 13 mV/g and a bandwidth of 12 Hz were achieved [39]. After that, oil and water were used in their accelerometers in 2011, the accelerometer was fabricated directly on an SU-8 organic substrate based on PCB, which ensured a high level of thermal isolation, and the sensitivity was improved to 32 mV/g [9]. 

In 2003, Mailly et al. from University Montpellier 2 presented a micromachined thermal accelerometer using a platinum heater and thermistors. Using air under a pressure of 25 bars, the sensor presented a high sensitivity of 116 mV/g (gain 2000), an equivalent acceleration noise of 0.3 mg RMS and a bandwidth of 20 Hz [19]. The influences of several parameters (nature and pressure of gas, cavity volume, the dimensions of the detectors) on the sensitivity or bandwidth of the thermal accelerometer were studied [21,23]. To improve the bandwidth (120 Hz as reported) without sensitivity decrease, a close-loop conditioning circuit was proposed, by which the bandwidth was improved to 1025 Hz [43]. Meanwhile, the linearity of the thermal accelerometer was also investigated. By optimizing the cavity dimension, the layout of the temperature detectors in the cavity and the electrical power on the heater, the thermal accelerometer was able to measure high-g accelerations in a range of 10,000 g with a linearity error lower than 4% [11].

#### 3.1.2. Tri-Axis Micromachined Thermal Accelerometers

With the advancement of micromachining technology, tri-axis thermal accelerometers emerging in 2008 have evolved for about ten years. In-plane accelerations can be easily measured by planar structures as the uniaxial or dual-axis accelerometers mentioned above, while out-of-plane acceleration measurement is a great challenge because it usually need tridimensional structure. 

**Figure 5:**
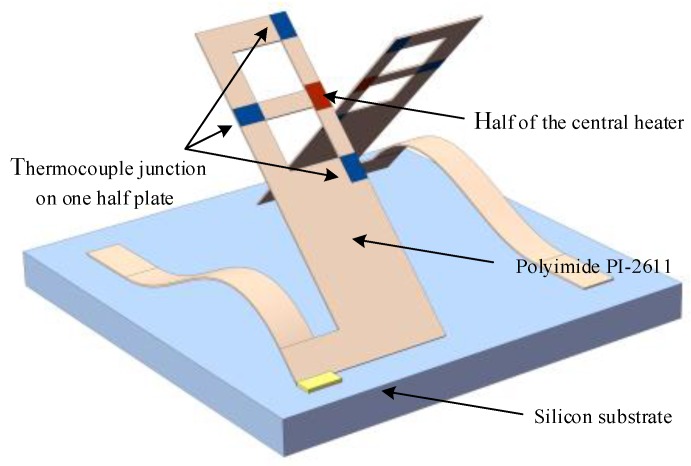
Illustration of tri-axis thermal accelerometer based on buckled cantilever microstructure.

In 2008, Leung et al. reported tri-axis thermal accelerometers fabricated using polyimide PI-2611 and assembled using a standard wire-bonding [46]. Compared with other thermal accelerometers, the proposed accelerometers fabricated entirely using polymer surface-micromachining technology did not need a large cavity, and instead, they used out-of-plane structures assembled during wire-bonding to provide thermal isolation as well as acceleration-sensitivity in *Z*-axis. In 2011, an improved buckled cantilever accelerometer (illustrated in Figure 5) was proposed by Leung’s group, including two half sensor plates attached to buckle cantilevers to form out-of-plane structures. Using a total heater power of 2.5 mW, the *X*, *Y*, and *Z* axes showed the sensitivities of 66, 64, and 25 μV/g respectively [47]. 

In 2011, Rocha et al. from University of Minho proposed a polymeric tri-axis thermal accelerometer based on the combination of flexible micromachining technology with microinjection molding [50]. The technologies they used brought the sensor design flexibility and freedom, but the performance of the accelerometer was poor, showing a sensitivity around 8 mV/g in *X* and *Y* axes and a sensitivity of 2.2 mV/g in *Z*-axis with a gain of 1000 [52]. 

Tridimensional structures are necessary for the tri-axis micromachined thermal accelerometers mentioned above, which are not compatible with the mature micromachining technology and the standard CMOS process. The misalignment during assembly is the key problem to be dealt with. Especially, for the buckled cantilever accelerometer, the complex and flimsy structure made it less robust and less reliable. 

**Figure 6:**
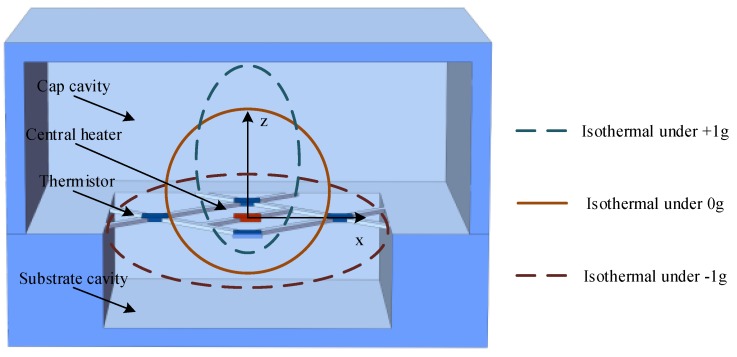
Structure and isothermals under different *Z*-axis accelerations of the planar tri-axis thermal convective accelerometer.

A feasible micromachined monolithic tri-axis accelerometer design was reported in 2013 by MEMSIC [105]. The reported tri-axis accelerometer had a planar structure similar to the dual-axis accelerometers, consisting of four groups of heater and thermopiles suspended in a silicon cavity, using the same principle to measure two in-plane accelerations. To measure the out-of-plane acceleration, it required an asymmetrical structure in up/down direction, which was accomplished by the implement of a larger cap cavity and a smaller cavity in the substrate. The thermal bubble was asymmetrical in up/down direction, the isotherm in the working plane changed with the presence of out-of-plane acceleration which was detected by the thermopiles and used to deduce the acceleration. Moreover, a tri-axis product was commercialized by MEMSIC but the sensing principle along the vertical axis was rarely reported [106]. Mailly from Montpellier University proposed a similar tri-axis thermal convective accelerometer using only one central heater (illustrated in Figure 6). The in-plane resolution reached 2.6 mg, suitable for most of consumer applications, while the out-of-plane resolution was only 60 mg [107]. 

These planar tri-axis thermal convective accelerometers are compatible with standard CMOS processes, and ASIC can be easily co-integrated on the same die with the sensors, which is very important for their commercialization.

**Figure 7:**
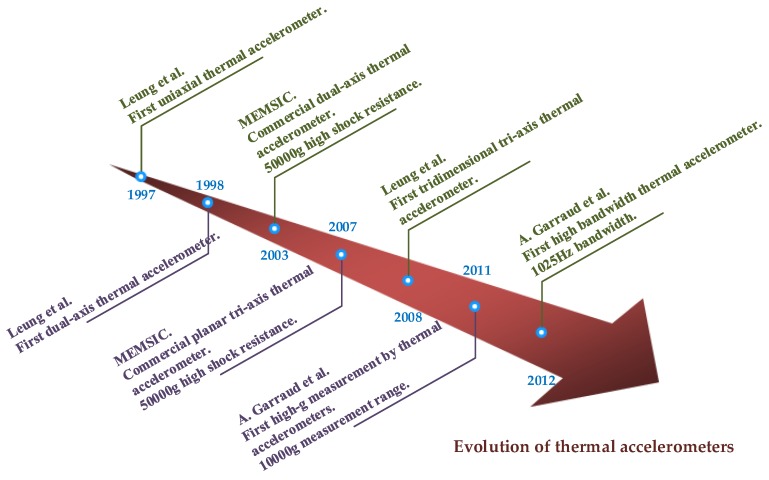
A brief chronology of the evolution of micromachined thermal accelerometers.

A brief chronology of the evolution of micromachined thermal accelerometers is illustrated in Figure 7, and typical research results of micromachined thermal accelerometers are summarized in Table 1. 

### 3.2. Micromachined Fluid Gyroscopes

As mentioned above, the micromachined fluid gyroscopes can be categorized into two types based on their driving principles, including jet flow gyroscope and thermal gas gyroscope. The jet flow gyroscope uses a laminar gas flow driven by micro pump, while the thermal gas gyroscope uses the thermal flow generated by thermal convection or thermal expansion 

#### 3.2.1. Micromachined Jet Flow Gyroscope

**Figure 8:**
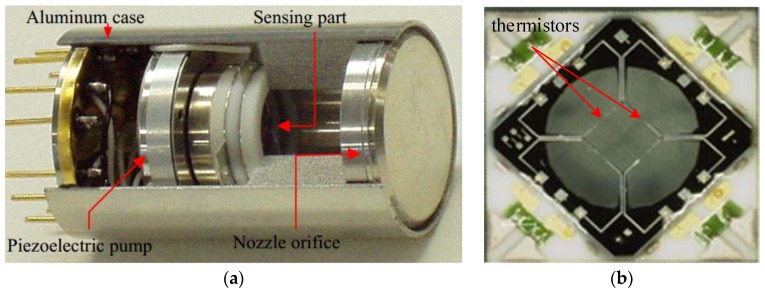
(**a**) Cut view of the dual-axis micromachined jet flow gyroscope; (**b**) micro graph of the sensing element [64].

Jet flow gyroscope emerged in 1960s and has been commercialized for military applications. For miniaturization of the gyroscopes, Shiozawa et al. from Tamagawa Seiki Co., Ltd (Nagano, Japan) and Dau et al. from Ritsumeikan University developed a dual-axis jet flow gyroscope (Figure 8) using micromachining and reported the work in 2004. The gyroscope consisted of a piezoelectric pump and a micromachined sensing element containing four thermistor wires, all of which were assembled in an aluminum case. Inert neon gas was used as the fluid medium in the device for its high thermal conductivity. The piezoelectric diaphragm pump was oscillated at a frequency of 7 KHz, creating a continuous gas flow with a peak flow velocity of 3.5 m/s at sensing element. The thermistors made of lightly-doped p-type silicon with a TCR of 4500 ppm/°C were set to about 50 °C higher than the ambient temperature [64]. Since then, their researches have been involved into optimization of nozzle orifice structure, shape and materials of the thermistors for the jet flow gyroscope. By adding sub-nozzles, the laminar jet flow regime at lower flow velocity was stabilized, and the sensitivity was increased up to 22%. By using T-shape thermistors, the thermal-induced stress was reduced 90.8% [65]. In addition, to further improve the sensitivity, the p-n junction thermistor with very high TCR was used instead of p-type silicon one [66]. They presented an improved gyroscope with a resolution of 0.5°/s and a bandwidth of 65 Hz in 2006 [65].

**Figure 9:**
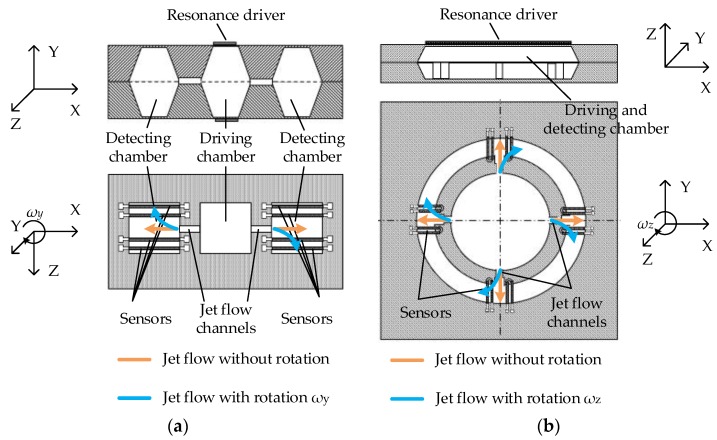
Monolithic uniaxial jet flow gyroscopes, (**a**) using three micro chambers [63]; (**b**) using only one chamber [111].

The above dual-axis micromachined jet flow gyroscopes still have large sizes and obvious alignment errors because of the assembly limitations. Monolithic integration is necessary to address these issues. Ding and Zhu et al. from Tsinghua University proposed a micromachined monolithic jet flow gyroscope design using three micro-chambers with a diaphragm pump and nozzle orifices as shown in Figure 9a, the patent of which was applied in 2001 and issued in 2004 [63]. In 2004, they further proposed a compact structure for the monolithic jet gyroscope by using one chamber with a diaphragm pump and cross-shaped micro channels to generate jet flows as shown in Figure 9b [111].

**Figure 10:**
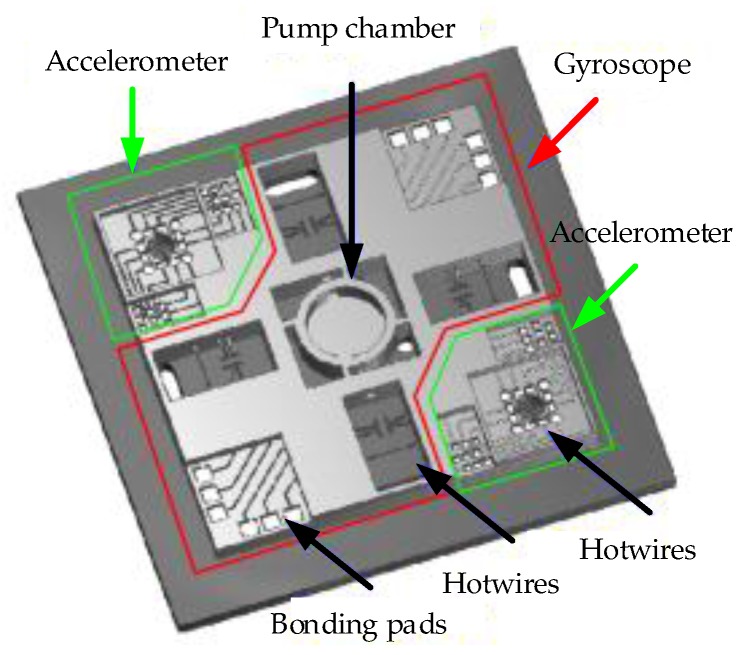
Monolithic jet flow inertial sensor consisting of tri-axis gyroscope and dual-axis accelerometer [82].

In 2005, Zhou from Peking University reported a design of monolithic uniaxial jet flow gyroscope [74], and in 2006 and 2007, Luo from HUST proposed another two similar designs [75,76], but no intensive study was reported after that. The research group from Ritsumeikan University reported a fully integrated MEMS-based jet tri-axis gyroscope in 2007 [78], and further integrated thermal convection accelerometers with the gyroscope [80,81,82], which could measure tri-axis rotations and dual-axis accelerations (shown in Figure 10). After that, their works focused on the optimization of the integrated PZT diaphragm pump for better stability and higher gas velocity, however, only a velocity of 1 m/s was achieved [88], which restricted the improvement of the monolithic jet flow gyroscopes.

In 2012, the Chang research group from Northwestern Polytechnical University proposed a micromachined jet flow gyroscope, called vortex gyroscope [112]. The vortex gyroscope used two opposite outer air pumps to drive gas flow which was rectified by a circular cavity to form a vortex gas flow. The flow velocity in the nozzle orifice could reach 5 m/s. Silicon thermistors were used to detect the deflection of the flow field. Thermistors fabricated by micromachining process on SOI wafer were glued on PCB board, and assembled with PMMA detection chamber. The gyroscope could measure tri-axis angular rotations and tri-axis accelerations with obvious cross-axis sensitivity. The gyroscope exhibited the rotation sensitivity of 0.642, 0.528, and 0.241 mV/°/s respectively, and a nonlinearity of 2.1%, 3.8%, and 4.5% respectively for *X*-axis, *Y*-axis and *Z*-axis [113].

#### 3.2.2. Micromachined Thermal Gas Gyroscope

Thermal gas gyroscopes use thermal convection or thermal expansion gas flow driven by the electrically-heated heaters, and use symmetrically-distributed thermistors to detect the change of the temperature distribution induced by the rotation. In thermal convection, fluid motion is generated by density differences in the fluid (buoyancy) occurring due to temperature gradients. While, in thermal expansion, fluid motion is generated by the fluid volume expansion in response to a change in temperature [97]. Compared with the jet flow gyroscopes, the sensor structures of the thermal gas gyroscopes are much simpler, but the sensitivity is lower because the thermal induced flow is slower than the jet flow. 

The first micromachined thermal gas gyroscope was proposed by Ding and Zhu et al. in 2001 [93]. In 2005, Zhu et al. prototyped the sensor based on convection heat transfer which could measure uniaxial angular rate and dual-axis accelerations simultaneously in one chip [71]. The sensor measured temperature gradients induced by the Coriolis acceleration acting on a gas flow sealed in a micro chamber. The sensor used only one heater and a pair of platinum thermistors. With a heater power about 14 mW, the sensitivity of the angular rate was about 20 μV/°/s with a gain of about 36,000, and the sensitivities for X and Y accelerations were about 430 and 256 mV/g with a gain of about 1500, respectively [72]. The sensor showed merits of low cost, wide working range (>1000°/s), and extremely high shock resistance (>20,000 g) [13]. In 2010, a theoretical characterization of the sensor incorporating its signal conditioning using a system level simplified model of a spring-damping system was proposed and experimental verification was demonstrated [73]. The modeling approach relied on the fundamentals of fluid mechanics and heat transfer, in association with empirical techniques. It was concluded that the nonlinearity was mainly attributed to the variable equivalent stiffness of the sensor system (i.e., a coefficient of the simplified equivalent model of a spring-damping system for the thermal gas gyroscope) and the structural asymmetry due to nonideal fabrication [73]. Moreover, the noise density of the sensor was measured to be 1°/s/Hz^1/2^. 

**Figure 11:**
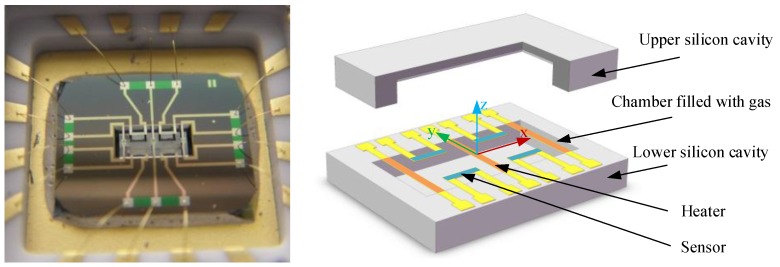
A thermal gas inertial sensor based on thermal expansion.

**Figure 12:**
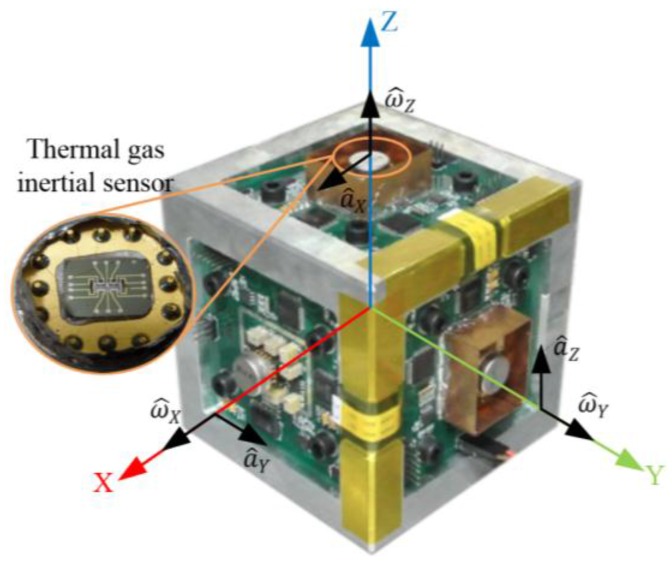
An MIMU using three gas inertial sensors based on thermal expansion [98].

In 2013, the group of Zhu reported an improved inertial sensor based on gas thermal expansion (shown in Figure 11) [96]. The sensor consisted of three alternatingly-heated heaters and two pairs of platinum thermistors symmetrically placed between the center and side heaters, and used gas movement based on heat expansion-contraction rather than heat convection to restrict the rotation-acceleration cross-coupling. The sensor could simultaneously measure uniaxial angular rate (±3000°/s, the nonlinearity <0.57%) and uniaxial acceleration (±10 g) in one chip by using a differential operational circuit, and demonstrated low cross-coupling effect via using thermal expansion flow instead of buoyancy-based convection flow. The sensor demonstrated an angular rate sensitivity of about 95 μV/°/s with a gain of 36,000 and an acceleration sensitivity of 300 mV/g with a gain of 10,000. A theoretical analysis on the thermal inertial sensor was conducted, where thermal expansion flow and convection flow were comprehensively interpreted for inertial measurements [97]. It was concluded that the inertial sensor based on heat convection suffered a strong cross-coupling effects between the rotation and acceleration measurements, while the sensor based on thermal expansion could restrained the cross-coupling due to its independence of buoyancy. In 2016, a MIMU using only three thermal-expansion-based inertial sensors shown in Figure 12 was developed by Liu and Zhu [98]. The measurement ranges of three-axes angular rates and three-axes accelerations reached ±4000°/s and ±10 g respectively, and the nonlinearity is less than 2% [98]. Besides, the system measurement errors of tri-axis angular rates and tri-axis accelerations were dramatically reduced by about two orders of magnitude using a neural-network-based compensation algorithm, which reached about 0.5°/s RMS for rotation and 1 mg RMS for acceleration, respectively.

Another design of thermal gyroscope was reported in 2012 by Leung et al. [68]. The gyroscope consisted of two heaters and a pair of platinum thermistors. Seismic gas flow was generated by alternately heating the two heaters, and the temperature differences caused by Coriolis force were detected by the pair of thermistors. Using SF_6_ gas at 101 kPa and a heater power 20 mW, the gyroscope exhibited a sensitivity of 0.947 mV/°/s with a gain of 18,742, a bandwidth of 40 Hz, and linearity better than 0.1% in the measurement range of ±1260°/s. In addition, the gyroscope was validated by the drop shocks of 2722 to 16,398 g [70]. Typical research results of micromachined fluid gyroscopes are summarized in Table 2.

## 4. Key Technologies of Micromachined Fluid Inertial Sensors

### 4.1. Micromachined Thermal Accelerometers

Some key technologies of micromachined thermal accelerometers related to bandwidth, temperature compensation, out-of-plane performance, test and calibration are discussed in this section.

#### 4.1.1. Bandwidth

**Figure 13:**
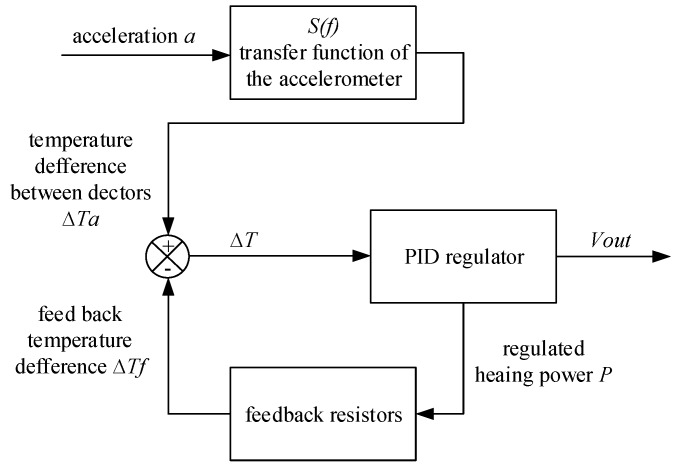
Block-diagram overview of the closed-loop configuration.

A major drawback of micromachined thermal accelerometers is their low bandwidth compared with solid seismic mass-based accelerometers [43]. The bandwidth was investigated by a research group from University Montpellier 2. They found that the bandwidth increased with the use of larger gas thermal diffusivity and smaller cavity. In 2008 the bandwidth reached 120 Hz by downsizing the cavity and thermistors [20] and was further improved to 320 Hz by using an gas with high thermal diffusivity (helium) and working pressure (2.15 bar) in 2011 [24]. However, as a matter of fact, a smaller cavity resulted in smaller sensitivity and the product of sensitivity and bandwidth was inversely proportional to the gas thermal diffusivity [24]. Therefore, a larger bandwidth is inconsistent with larger sensitivity and it is necessary to find other solutions to improve the bandwidth without decreasing the sensitivity. In 2012, they proposed a closed-loop sensor configuration with two additional resistors placed closely to temperature detectors to rebalance their temperatures (illustrated in Figure 13) [43]. The scheme was to keep the detector temperature constant to decrease the overall response time, and the closed-loop bandwidth was improved to 1025 Hz compared with an open-loop bandwidth of 66 Hz, all of which were measured with nitrogen at atmospheric pressure. 

#### 4.1.2. Temperature Compensation

The performance of the thermal accelerometer depends on the ambient temperature due to its thermal sensitivity. A simple model was developed by Leung et al., which suggested that the output of thermal accelerometer is linearly proportional to the Grashof number, *Gr* (formulated in Equation (1)) [14,15]:

(1) $$Gr=\frac{a\rho^{2}\beta \Delta Tl^{3}}{\mu^{2}}$$

where *a* is acceleration, *ρ* is gas density, *β* is coefficient of expansion of gas, *μ* is gas viscosity, *ΔT* is temperature difference between the heater and the ambient, and *l* is linear dimension. 

Because *ρ* and *β* are inversely proportional to the ambient temperature *T*^−1^, while *μ* is proportional to *T*^1/2^ for an ideal gas, it can be concluded that the sensitivity decreases with the ambient temperature. Experimental results showed that temperature dependence of the sensitivity was not linear [19,105]. A temperature compensation method was proposed by MEMSIC for its commercial thermal accelerometer, which used a gain adjustment of 0.9%/°C to keep the sensitivity error within 5% of its room temperature value [114]. 

#### 4.1.3. Out-of-Plane Performance of Planar Tri-Axis Micromachined Thermal Accelerometers

As mentioned in Section 3.1.2, two kinds of monolithic tri-axis thermal accelerometers have been developed: the tri-axis ones and the planar ones. The tri-axis accelerometers were less robust and reliable due to their out-of-plane structures. What’s more, they were not compatible with the mature micromachining and the standard CMOS process, which limited their batch fabrication. The planar ones showed advantages of easy fabrication, and have been commercialized. However, all planar tri-axis accelerometers reported up to now have common drawback that the sensitivity of the out-of-plane acceleration was much smaller than that of the in-plane acceleration, resulting in a poor out-of-plane resolution [105,107]. It is important to improve out-of-plane performance for more competitive planar tri-axis accelerometers. 

#### 4.1.4. Test and Calibration Strategy for Batch Fabrication

In the past decades, a number of MEMS devices have been demonstrated and widely used in many applications, which benefit from the batch manufacturing capabilities of micromachining. A satisfactory production yield is the key issue to maintain low production costs. The application of physical stimuli is usually required to verify their parameters, which necessitates specific and sophisticated equipment that is much more expensive than standard electrical test equipment [59]. Therefore, alternative test method based on electrical test stimuli is necessary to facilitate testing and reduce its cost. It is necessary as well to find a calibration strategy, which is used to improve production yield by compensating the susceptibility of device performance to process scattering. 

The research groups from University Montpellier 2 and University of Sfax have conducted relevant researches on a commercial thermal accelerometer for many years. Firstly, they established an accurate behavioral model of the accelerometer, which could be used to evaluate the influences of structure parameters, especially the cavity depth that was quite dispersive for etch process scattering, on the conductive and convective phenomenon [49]. The established compact model permitted fault injection and supported Monte-Carlo simulations, which would be extremely time-consuming in FEM since it required 3D simulations involving both solid and fluidic elements [58]. Afterwards, based on Monte-Carlo simulations, it was demonstrated that the relative deviation of the equivalent impedance of the Wheatstone bridge at ambient temperature and under nominal biasing conditions had a rather good correlation with the differential temperature resulted from an acceleration, and therefore influenced the system sensitivity [60]. An effective electrical test method of the accelerometer sensitivity was proposed by testing the relative deviation of the equivalent impedance of the Wheatstone bridge. Meanwhile, a calibration strategy was proposed, and was used to compensate the inconformity of accelerometer sensitivity caused by process divergence via adjusting the heater resistor biasing voltage of each device. The calibration strategy was implemented by an on-chip programmable biasing voltage based on an integrated PWM or PDM generator [60]. As a consequence, a production yield of more than 99.8% could be obtained for low-cost products using only electrical tests for the calibration scheme [61]. Modelling method was further studied to improve the behavioral model accuracy of accelerometer [28,53,54,55], and the electrical calibration strategy was improved for its better robustness [62].

### 4.2. Micromachined Fluid Gyroscopes

The technologies of micromachined fluid gyroscopes are less mature than that of the thermal accelerometers. Therein, the key technologies including device integration, thermal compensation, and cross-coupling compensation are mainly investigated, which are discussed in this section.

#### 4.2.1. Integration of Micro Jet Gyroscope

**Figure 14:**
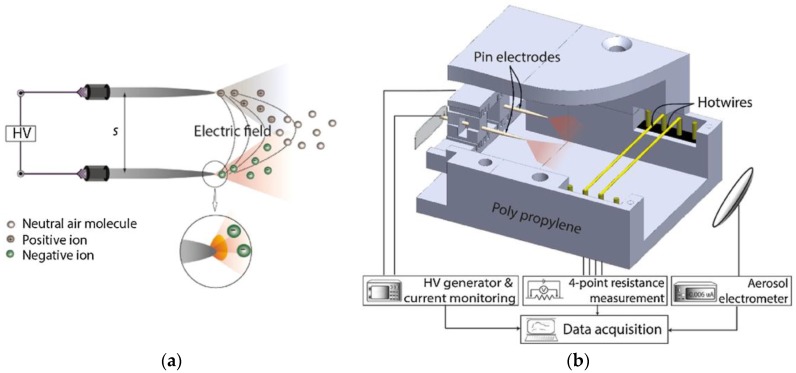
(**a**) Schematic view of bipolar corona discharge enhanced air flow; (**b**) Schematic design of device and measurement setup: a battery operated high voltage generator is connected to parallel pin electrodes and the ion wind is measured by hotwires heated by constant current [91].

For the micromachined jet flow gyroscopes, monolithic integration is necessary to minimize the size and the alignment error induced by the assembly process. Comparatively, the integration of micro-pumps is more challenging. The research groups from Ritsumeikan University and Sumitomo Chemical Ltd have worked on improving the integration of the micro-pump in their jet gyroscopes for about 10 years. The structures of nozzle, flow channel, pumping and working chambers were optimized, and a gas flow velocity of 1 m/s was obtained in 2015 [88]. To enhance the gas flow velocity, a method using bipolar corona discharge based air flow was reported in 2016 [90,91,92]. Shown in Figure 14a,b, the corona discharge was operated to generate both positive and negative ions using two sharp electrodes placed in parallel, and the ion winds originated from both electrodes assisted the bulk flow moving along the center of the electrode interspace, which resulted in a boosted jet without charging the flow. The corona discharge helped to focus the jet and increased the flow peak velocity from 1.41 m/s to 2.42 m/s with a power of 27.1 mW [90].

#### 4.2.2. Thermal Compensation

In the study of Ritsumeikan University in 2008, a temperature dependence of the micromachined jet flow gyroscope sensitivity was measured to be 0.11%/°C for the *X*-axis and 0.16%/°C for the *Y*-axis. These unwanted characteristics were removed by adjusting the amplifier with a temperature resistor, and the dependence of the gyroscope sensitivity on the ambient temperature was reduced to be 0.02%/°C for both axes [67].

Similar to the thermal accelerometers, the performances of the thermal gas gyroscopes depend on the ambient temperature as well. In 2015, Liu and Zhu presented a self-compensation technology to overcome the temperature drift for the thermal gas gyroscopes [103]. The compensation methodology utilized an alternating constant-temperature-difference (CTD) operation circuit to thermally drive gas motion for stabilizing the sensor sensitivity and reducing the temperature drift. The proposed method could realize self-sustained temperature compensation through an analogy circuitry, which was easily operated and with good stability. Experimental results validated that the temperature dependences of the sensitivity and baseline of the thermal gas gyroscope were effectively reduced to about 20% and 25% of uncompensated values, respectively [103].

#### 4.2.3. Cross Coupling Error Compensation

The cross coupling effect of the thermal gyroscope is caused by the complex thermal fluidic motion in the sensor chamber. It has been theoretically demonstrated that thermal convection flow generally exists in a fluid dynamic system, that unavoidably results in a cross coupling effect between acceleration and rotation measurements [98]. Although an impressive decrease of the coupling effect was obtained via using thermal expansion instead of thermal convection [97], the cross coupling errors could not be ignored because the thermal convection flow generally exists. 

Leung et al. proposed a self-compensation method [102]. By using a switching frequency of the heaters, the thermal gas gyroscope could be operated at transient and steady states. In the transient sate, the sensor worked as a gyroscope, while, in the steady state, it worked as a thermal convection accelerometer. Therefore, the acceleration signal obtained in the steady state could be used to compensate the gyroscope output. However, the bandwidth of the thermal gyroscope was greatly reduced and limited by this method.

Liu and Zhu presented a thermal gas MIMU based on thermal expansion and used a neural network based data fusion to compensate the cross-coupling errors of the MIMU. Through a comprehensive calibration, the errors mainly caused by cross coupling and misalignment could be compensated. Experiments validated that the measurement errors of tri-axis angular rates and tri-axis accelerations of the MIMU were reduced by almost two orders of magnitude [98].

## 5. Conclusions

In this review, the working principles of micromachined fluid inertial sensors including micromachined thermal accelerometers and fluid gyroscopes are introduced and the technology evolution is reviewed comprehensively. The micromachined thermal accelerometers based on thermal convection have been developed maturely and become commercialized. The micromachined fluid gyroscopes are mainly based on jet flow and thermal flow induced by thermal convection or expansion. Compared with jet flow gyroscopes, the thermal inertial gyroscopes have relatively simple structures compatible with monolithic integration. Key technologies of thermal accelerometers are discussed, including bandwidth, temperature compensation, out-of-plane performance of planar tri-axis accelerometers, and test and calibration strategy for batch fabrication aiming at improving their performances and production yields, decreasing their costs, and enhancing their competitiveness in the market. The key technologies of the micromachined fluid gyroscopes mainly involve improving the integration and sensitivity, reducing the thermal errors and cross coupling errors, which must be overcome before commercialization. In general, micromachined fluid gyroscopes are less mature than thermal accelerometers. Up to now, the micromachined fluid inertial sensors still face the challenges of low sensitivity, small bandwidth, susceptibility to ambient temperature, etc. Nevertheless, the prospects are bright due to their inherent advantages of low cost, small size, batch fabrication and high shock resistance. 

## Figures and Tables

**Table 1:** Typical research results of thermal fluid accelerometers.

Year	Research Institute	Structure	Fluid	Sensitivity	Resolution/Noise	Bandwidth	Measurement Range	Shock Survival	Reference
1997~2012	Simon Fraser University, Burnaby, BC, Canada	uniaxial	Air, isopropanol	7 V/g	0.6 mg	20 Hz	±1 mg~1 g		[7,14,16]
1998	Simon Fraser University	dual-axis	air		0.6 mg	20 Hz			[15]
1998	George Washington University, Washington, DC, USA	uniaxial		185 µV/g ^1^		600 Hz ^1^	0~7 g		[36]
				115 µV/g ^2^		100 Hz ^2^			
2001	Hebei Semiconductor Research Institute, Shijiazhuang, China	uniaxial		600 µV/g	1 mg·Hz^−1/2^	75 Hz	10 g		[29]
2002	HSG-IMIT, Villingen-Schwenningen, Germany	uniaxial	SF_6_	6.6 mV/°	0.003°				[6]
2003~2016	MEMSIC, Wuxi, China	uniaxial & dual- axis	air	1 V/g	0.4 mg RMS	160 Hz	±1~100 g	50,000 g	[104,108,109]
2003~2011	University Montpellier 2, Montpellier, France	uniaxial	Air, CO_2_, helium	58 µV/g	0.3 mg RMS	320 Hz	0~3 g		[19,20,21,23]
2004~2011	TEI of Athens, Athens, Greece	uniaxial	Air, water	32 mV/g		12 Hz			[9,39]
2006	Ritsumeikan University, Kyoto, Japan	dual-axis		13 mV/g			±5 g		[38]
2007~2016	MEMSIC, Wuxi, China	tri-axis	xenon	0.5 V/g	2.5 mg RMS	17 Hz	±8 g	50,000 g	[105,106,110]
2008~2011	Simon Fraser University	tri-axis	SF_6_	*XYZ*: 66, 64, 25 μV/g			±1 g		[45,47]
2011	University Montpellier 2	uniaxial	gas				10,000 g		[11]
2012	University Montpellier 2	uniaxial	nitrogen	0.034 °C/g		1025 Hz			[43]
2014	University Montpellier 2	tri-axis	air		*XY*: 2.6 mg, *Z*: 60 mg	20 Hz			[107]
2015	University of Minho, Braga, Portugal	tri-axis	air	*XY*: 8 µV/g, *Z*: 2.2 µV/g		4 Hz ^3^			[52]

^1^ Using thermistors as temperature sensors; ^2^ Using thermocouples as temperature sensors; ^3^ Based on simulations.

**Table 2:** Typical research results of micromachined fluid gyroscopes.

Year	Research Institute	Working Principle	Sensitivity	Resolution	Measurement Range	Shock Survival	Reference
2001~2016	Tsinghua University, Beijing, China	Thermal gas MIMU	95 μV/°/s (Gain 36,000)	0.5°/s 1 mg	±4000°/s ± 10 g	>20,000 g	[13,71,72,73,93,94,95,96,97,98]
			300 mV/g (Gain 10,000)				
2004~2016	Ritsumeikan University, Kyoto, Japan	Jet flow	*X*: 0.082 mV/(°/s)	0.5°/s			[65]
			*Y*: 0.078 mV/(°/s)				
2010~2014	Simon Fraser University, Burnaby, BC, Canada	Thermal gas	0.947 mV/°/s (Gain 18,742)		±1260°/s	16,398 g	[70]
2012~2015	Northwestern Polytechnical University, Xi‘an, China	Vortex jet flow	*X*: 0.642 mV/°/s	*X*: 0.04°/s ^1^	±100°/s		[112,113]
			*Y*: 0.528 mV/°/s	*Y*: 0.05°/s ^1^			
			*Z*: 0.241 mV/°/s	*Z*: 0.2°/s ^1^			

^1^ Theoretical value.
